# Serum CXCL10/IP-10 may be a potential biomarker for severe *Mycoplasma pneumoniae* pneumonia in children

**DOI:** 10.1186/s12879-021-06632-4

**Published:** 2021-09-04

**Authors:** Mengyao Li, Ying Chen, Huihan Li, Dehua Yang, Yunlian Zhou, Zhimin Chen, Yuanyuan Zhang

**Affiliations:** 1 Department of Pulmonology, Children’s Hospital, Zhejiang University School of Medicine, National Clinical Research Center for Child Health, Hangzhou, China; 2 Department of Pediatrics, Ningbo Medical Center Lihuili Hospital, Ningbo, China

**Keywords:** CXCL10/IP-10, Prediction, *Mycoplasma pneumoniae* pneumonia, Children

## Abstract

**Background:**

How to early distinguish the severity of *Mycoplasma pneumoniae* pneumonia (MPP) is a worldwide concern in clinical practice. We therefore conducted this study to assess the relationship between levels of serum inflammatory chemokines and the severity of MPP.

**Methods:**

In this prospective study, we enrolled 39 children with MPP, whose clinical information was collected, blood samples were assayed for cytokines and chemokines by ELISA.

**Results:**

The levels of serum CXCL10 in children with severe MPP were significantly higher than those in children with mild MPP (2500.0 [1580.9–2500.0] vs. 675.7 [394.7–1134.9], P < 0.001). Measurement of CXCL10 levels in serum enabled the differentiation of children with severe MPP with an area under the curve (AUC) of 0.885 (95 % CI 0.779–0.991, P < 0.001), with a sensitivity of 81.0 % and a specificity of 83.3 %.

**Conclusions:**

Serum CXCL10 level may be a potential biomarker for severe MPP in children.

## Background

*Mycoplasma pneumoniae* (MP) is among the smallest self-replicating bacteria that lack typical bacterial cell walls [1]. It is a common pathogenetic organism of respiratory infection in children. *Mycoplasma pneumoniae* pneumonia (MPP) accounts for approximately 8 to 40 % of community-acquired pneumonia (CAP) in children aging from 3 to 15 with regional epidemics occurring every 3 to 7 years [2, 3]. Although MPP spreads easily among children who are in close contact with each other, it is typically a self-limited disease. However, severe MPP happens with serious pulmonary and extrapulmonary complications at times, including pulmonary atelectasis, necrotizing pneumonia, myocardial damage and peripheral embolization, which may result in serious impacts on children’s clinical outcome and quality of life. Early diagnosis and prompt treatment are of great significance in reducing the mortality and sequelae of children with severe MPP. Therefore, there is an urgent need to identify some valid biomarkers for the severity of MPP.

Chemokines with four subfamilies: CXC, CC, C and CX3C, are involved in many biological processes including tumor growth and metastasis, inflammation, angiogenesis, and migration of immune cells [4]. Based on their function, chemokines can be categorized into inflammatory, homeostatic and dual-function chemokines, as well. Inflammatory chemokines, including CXCL10/IFN-γ-inducible protein-10 (IP-10), CCL2, CCL5, CCL8, are found to be elevated under inflammatory conditions and principally involved in the recruitment of leukocytes to inflammatory sites [5]. Recently, there has been increased emphasis on the vital links between inflammatory chemokines and infectious diseases. It has been observed that CCL5 was highly expressed in the serum of MPP patients and the level of it was positively related to MP-DNA [6]. In addition, CXCL10 was found to be critical for the control of T. gondii chronic infection in the eye by affecting the maintenance of the T-cell response [7]. However, little research to date has determined the role of inflammatory chemokines in severe MPP.

Therefore, this prospective study aimed to explore the relationship between the serum levels of inflammatory chemokines and the severity of MPP, which shed new light on novel biomarkers for children with severe MPP.

## Methods

### Study population

**Fig. 1:**
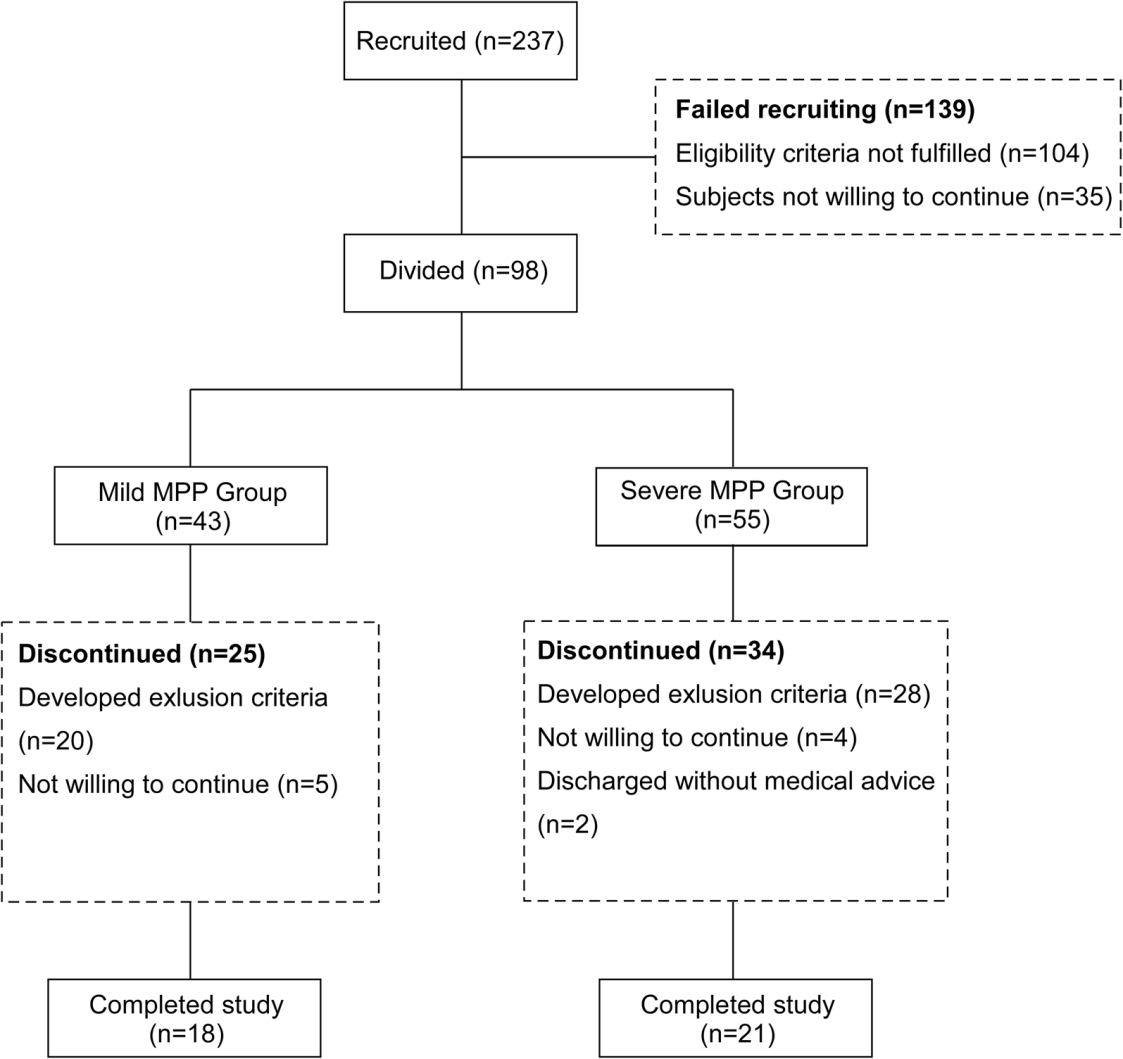
Flow chart of patient recruitment

From March 2013 to June 2014, 39 children with MPP from the Children’s Hospital, Zhejiang University School of Medicine were recruited for this prospective analysis. Criteria for selecting the subjects were as followings: (i) clinical presentation (fever, cough, tachypnea, abnormal breath sounds) and radiologic evidence of CAP (interstitial infiltrates, segmental and lobar consolidations, hilar lymph node enlargement); (ii) microbiological evidence from serologic testing, positive polymerase chain reaction (PCR) tests of nasopharyngeal secretions or bronchoalveolar lavage fluid (BALF), indications for bronchoscopy were persistent radiological abnormalities (atelectasis and consolidation of lung fields). Exclusion criteria were the followings: (i) patients with primary or secondary immune deficiency/dysfunction, including congenital heart disease, chronic liver or kidney disease, oncologic disorders, connective tissue disease, chronic lung disease; (ii) patients in convalescent-phase; (iii) patients with mixed infection; (iv) patients diagnosed with severe MPP later during the hospitalization as the disease progressed. The flow chart of patient selection was displayed in Fig. 1.

All the patients were categorized into mild MPP group (n = 18) and severe MPP group (n = 21), according to the clinical parameters and laboratory tests on admission. The severity of MPP was defined based on the criteria of community-acquired pneumonia [8–10]. The mild MPP group was defined as respiratory rate < 70 breaths/min at age < 3 years old or respiratory rate < 50 breaths/min at age ≥ 3 years old, no dehydration, normal food intake. Meanwhile, the severe MPP group was defined as tachypnea with respiratory rate ≥ 70 breaths/min at age < 3 years old or respiratory rate ≥ 50 breaths/min at age ≥ 3 years old (without interference from fever and cry), increased work of breathing (flaring of the nares, marked retractions, grunting), capillary refill time ≥ 2 s, cyanosis, anorexia and dehydration, appearance of pulmonary and extrapulmonary combinations including pleural effusion, lung necrosis/lung abscess, myocardial damage and peripheral embolization.

This study received ethical clearance from the Ethics Committee at Children’s Hospital, Zhejiang University School of Medicine. All methods were carried out in accordance with relevant guidelines and regulations. Parents or legal guardians of all the participants provided written informed consents.

### Detection of serum cytokines and chemokines levels 

Venous blood samples of each patient were collected on admission. Measurements of cytokines IL-10 and chemokines CXCL10, CCL2, CCL8 in serum were quantified by Human Inflammatory Cytokine ELISA Kit and Human Chemokine Kit (Becton, Dickinson and Company), according to the manufacturer’s standard protocol.

### Statistical analysis

Statistical analyses were performed with the SPSS software, version 20.0. Continuous variables were summarized as median (interquartile range) while categorical variables were described as proportion. Clinical characteristics that were significant (P < 0.1, univariate analysis) were included in the multivariate forward stepwise logistic regression analysis to identify independent influence factors. Diagnostic accuracy was estimated by the Receiver Operating Characteristic (ROC) curve analysis. Spearman rank correlations were used to assess correlations between variables. A P-value < 0.05 was considered as statistically significant.

## Results

### Clinical features of children with MPP

In all, 39 children with MPP from the Children’s Hospital, Zhejiang University School of Medicine were recruited into our study. All the patients had positive serology of MP. Among them, 36 children were confirmed with MPP using BAL while 3 children were confirmed by nasopharyngeal swabs because they didn’t meet the indications for BAL, which are mentioned in the Methods. Based on the criteria described in the Methods, they were separated into severe MPP group and mild MPP group. The clinical information for both groups is summarized in Table 1.

**Table 1:** Clinical information of children with MPP

Parameters	Severe MPP	Mild MPP
	**(n = 21)**	**(n = 18)**
Male (%)	42.9	66.7
Age (month)	73.0 (58.0–103.0)	72.0 (47.0–93.3)
Duration of fever before recruitment (d)	8.0 (7.0–9.5)	6.0 (4.5–7.0)
Duration of fever after recruitment (d)	7.0(5.0–10.5)	2.0 (0–4.0)
Length of stay (d)	15.0 (9.0–18.0)	6.5 (4.8–9.0)
Extrapulmonary manifestations (%)	33.3	0
WBC (×10^9^ cells/L)	7.5 (6.3–9.3)	6.5 (5.3–9.1)
Neutrophils proportion (%)	77.5 (72.3–85.5)	64.4 (53.5–70.0)
CRP (mg/L)	85.0 (45.5–156.0)	7.5 (3.0–17.0)
LDH (IU/L)	645.0 (546.0–922.5)	331.0 (261.0–412.8)
ALT (U/L)	41.0 (16.5–58.0)	15.0 (11.5–20.3)
AST (U/L)	55.0 (44.0–73.0)	31.5 (22.3–39.3)

*WBC* White blood cell count, *CRP* C-reactive protein, *LDH* Lactate dehydrogenase, *ALT* Alanine aminotransferase, *AST* Aspartate aminotransferase. Quantitative data are represented as median (interquartile range)

### Serum levels of chemokines in children with MPP

**Fig. 2:**
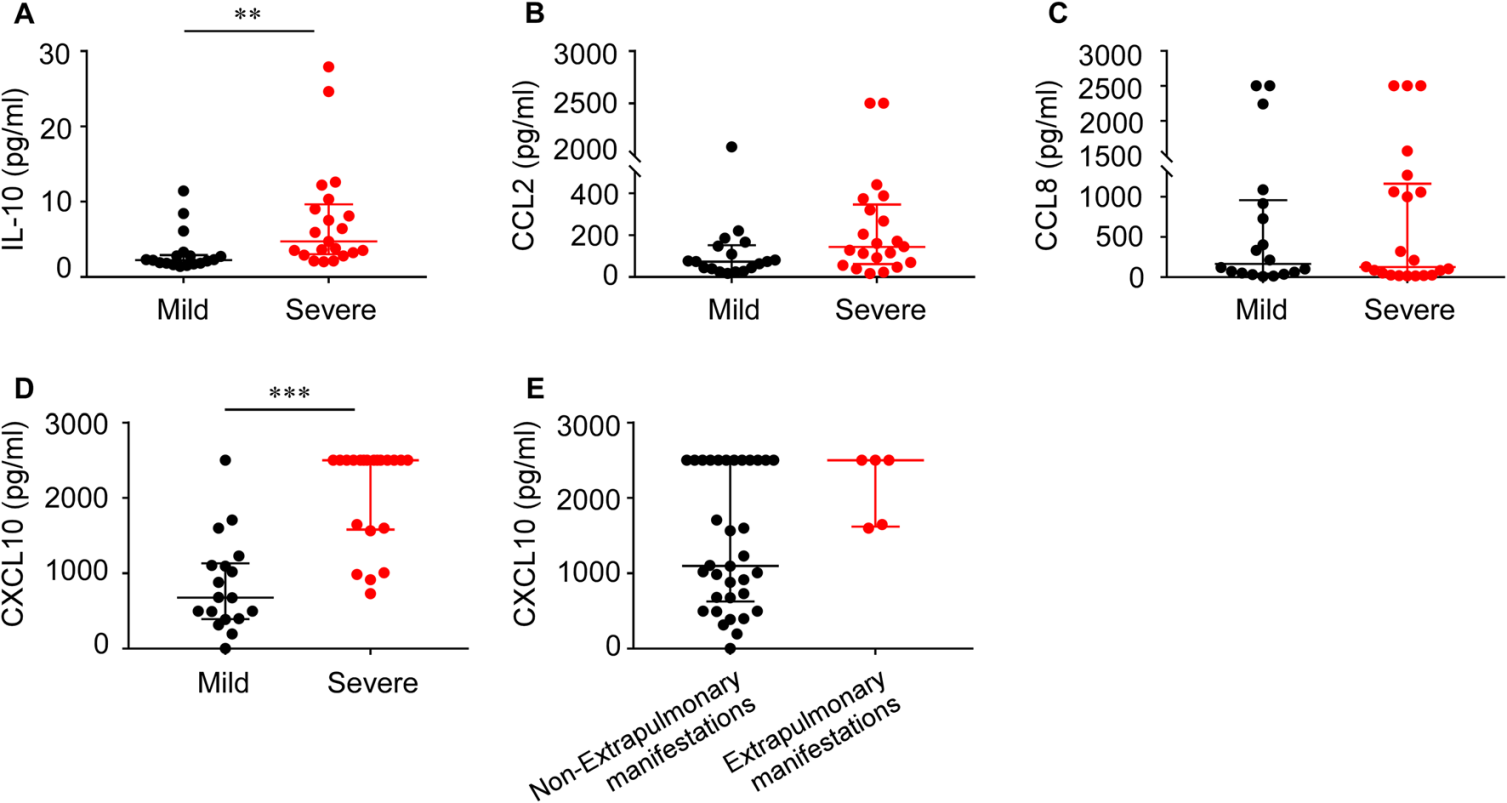
Serum levels of cytokines and chemokines in MPP children. Serum levels of IL-10 (**A**), CCL2 (**B**), CCL8 (**C**) and CXCL10 (**D**) in mild MPP and severe MPP children. (**E**) Serum levels of CXCL10 in children with and without extrapulmonary manifestations. Mann-Whitney *U* test, P < 0.05 considered statistically significant, **P < 0.01 and ***P < 0.001

Several recent studies have confirmed an association between some pro-inflammatory cytokines like IL-10 in serum and MPP infection [11, 12]. We further analyzed the levels of serum IL-10 and chemokines between the two groups. Figure 2 displays the median and the interquartile range of serum IL-10, CXCL10, CCL2, CCL8 levels from both groups. Compared to mild MPP group, the levels of serum CXCL10 in severe MPP group were significantly higher (2500.0 [1580.9–2500.0] vs. 675.7 [394.7–1134.9], P < 0.001), as well as IL-10 (4.7 [3.1–9.7] vs. 2.3 [1.8–2.9], P < 0.001). Nevertheless, there was no significant difference found in the serum levels of CCL2 and CCL8 between the two groups (P > 0.05). Moreover, there was no difference in CXCL10 levels between children with and without extrapulmonary manifestations (P > 0.05). A binary logistic regression analysis was further performed to identify that serum levels of CXCL10 and LDH were independent indicators for MPP severity (Table 2).

**Table 2:** Logistic regression analysis of clinical information associated with severe MPP

Variables	Univariate Logistic Regression			Multivariate Logistic Regression		
	**Coefficient**	**OR (95 %CI)**	**P–value**	**Coefficient**	**OR (95 %CI)**	**P–value**
Male	− 0.981	0.375 (0.102, 1.385)	0.14			
Age	0.013	1.014 (0.992, 1.036)	0.23			
Duration of fever before recruitment	0.380	1.462 (1.067, 2.003)	0.02*			
Duration of fever after recruitment	0.546	1.726 (1.227, 2.430)	0.002**			
Length of stay	0.417	1.518 (1.154, 1.997)	0.003**			
WBC	0.181	1.198 (0.911, 1.576)	0.20			
Neutrophils proportion	0.181	1.198 (1.060, 1.354)	0.004**			
CRP	0.048	1.049 (1.017, 1.083)	0.003**			
LDH	0.008	1.008 (1.003, 1.012)	0.002**	0.007	1.007 (1.001, 1.012)	0.002**
ALT	0.118	1.125 (1.030, 1.229)	0.009**			
AST	0.083	1.087 (1.024, 1.153)	0.006**			
IL–10	0.275	1.317 (1.016, 1.707)	0.04*			
CCL2	0.001	1.001 (0.999, 1.002)	0.35			
CCL8	0.000	1.000 (0.999, 1.001)	0.83			
CXCL10	0.002	1.002 (1.001, 1.004)	< 0.001***	0.002	1.002 (1.001, 1.004)	0.005**

*WBC* White blood cell count, *CRP* C-reactive protein, *LDH* Lactate dehydrogenase, *ALT* Alanine aminotransferase, *AST* Aspartate aminotransferase. P < 0.05 considered statistically significant, *P < 0.05, **P < 0.01 and ***P < 0.001

### Diagnosis value of CXCL10 in children with severe MPP

**Fig. 3:**
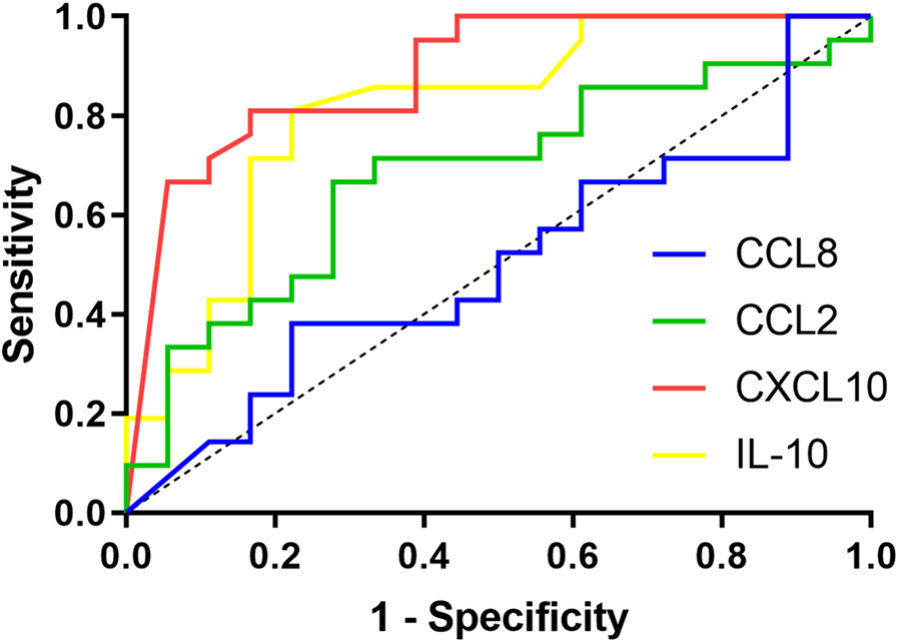
ROC curve of IL-10, CXCL10, CCL2, CCL8 for the identification of severe MPP

Next, we evaluated the ability of CXCL10 for recognition of severe MPP in children. ROC curve was performed. Optimum cutoff value was determined as well, which are presented in Table 3; Fig. 3. The area under the receiver operating characteristic curve (AUC) of CXCL10 was 0.885 (95 % CI 0.779–0.991, P < 0.001). With an optimal cutoff value of 1396.60 pg/ml, the sensitivity was 81.0 % and the specificity was 83.3 % for identifying severe MPP.

**Table 3:** ROC analysis of cytokines and chemokines for the identification of severe MPP

Parameters	AUC	Cutoff value	Sensitivity	Specificity	P–value	95 % confidence interval
IL–10 (pg/ml)	0.812	2.85	0.810	0.778	0.001**	0.672–0.952
CXCL10 (pg/ml)	0.885	1396.60	0.810	0.833	< 0.001***	0.779–0.991
CCL2 (pg/ml)	0.680	110.45	0.667	0.722	0.06	0.509–0.851
CCL8 (pg/ml)	0.503	953.70	0.381	0.778	0.98	0.317–0.688

P < 0.05 considered statistically significant, **P < 0.01 and ***P < 0.001

### Correlation between CXCL10 and clinical characteristics in children with MPP

**Fig. 4:**
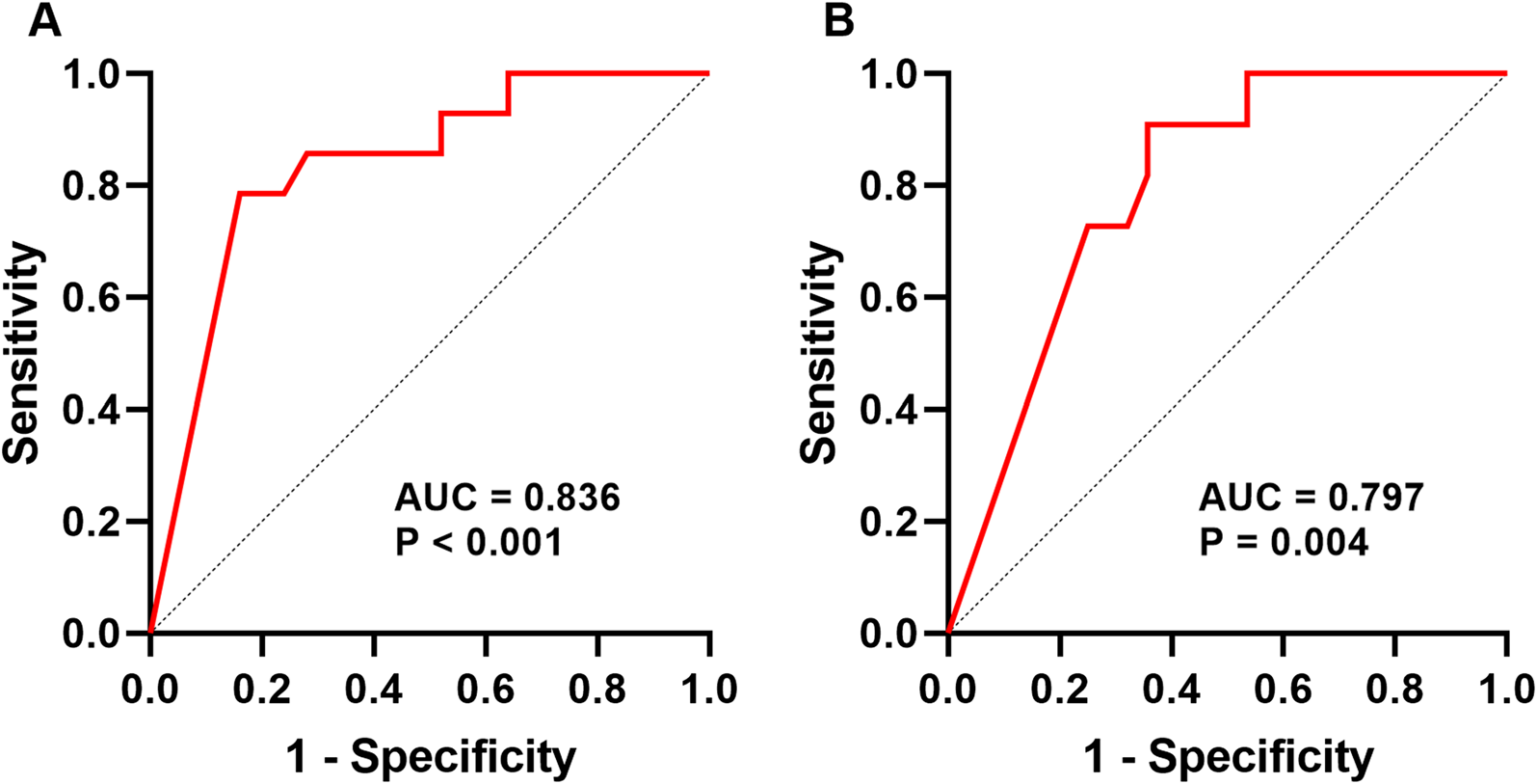
ROC curve of CXCL10 for predicting severe MPP-related clinical manifestations. ROC curve of CXCL10 for predicting duration of fever after recruitment (**A**) and length of stay (**B**)

As shown in Table 4, serum concentrations of CXCL10 were strongly associated with duration of fever (r = 0.706, P < 0.001), length of stay (r = 0.602, P < 0.001), CRP (r = 0.693, P < 0.001), ALT (r = 0.624, P < 0.001) levels. Moreover, they were moderately correlated with LDH (r = 0.498, P = 0.001), AST levels (r = 0.390, P = 0.01) and the proportion of neutrophils (r = 0.525, P = 0.001). However, no significant correlation was found between CXCL10 and WBC (P > 0.05). In addition, ROC curve analysis (Fig. 4) indicated that serum CXCL10 level of 2102.75 pg/ml predicted duration of fever after recruitment ≥ 7 days with a sensitivity of 78.6 % and specificity of 84.0 % (AUC = 0.836, P < 0.001), level of 1396.60 pg/ml predicted length of stay ≥ 15 days with a sensitivity of 90.9 % and specificity of 64.3 % (AUC = 0.797, P = 0.004).

**Table 4:** Correlation between serum CXCL10 and clinical characteristics in children with MPP

CXCL10	Spearman correlation	Correlation P–value
Duration of fever (d)	0.706	< 0.001***
Length of stay (d)	0.602	< 0.001***
WBC (×10^9^ cells/L)	0.109	0.51
Neutrophils proportion (%)	0.525	0.001**
CRP (mg/L)	0.693	< 0.001***
LDH (IU/L)	0.498	0.001**
ALT (U/L)	0.624	< 0.001***
AST (U/L)	0.390	0.01*

*WBC* White blood cell count, *CRP* C-reactive protein, *LDH* Lactate dehydrogenase. P < 0.05 considered statistically significant, *P < 0.05, **P < 0.01 and ***P < 0.001

## Discussion

Although the pathogenesis of MPP is not fully understood, accumulating evidence implicates that cytokines and other reactive substances play a critical role in this immune-mediated lung disease. It has been demonstrated that serum IL-10 and IFN-γ in refractory *Mycoplasma pneumoniae* pneumonia (RMPP) group were significantly higher than those in general *Mycoplasma pneumoniae* pneumonia (GMPP) group [13]. Meanwhile, CRP ≥ 16.5 mg/L, LDH ≥ 417 IU/L and IL-6 ≥ 14.75 pg/ml might be the significant predictors of RMPP in children [14]. However, chemokines, as important regulatory factors of leukocyte migration and activation, are also involved in the development of lung pathology in various acute and chronic lung diseases [15]. Previous research has shown that *Mycoplasma fermentans*-derived membrane component macrophage-activating lipopeptide 2 (MALP-2) could stimulate human monocytes to release IL-8, CCL2 and CCL3, which were also found to be significantly elevated in the BALF of mice after MP infection [16]. Nevertheless, compared with the procedure of BAL, the sampling of serum is more economical and easily accessible in clinical work.

CXCL10/ IP-10 is a CXC chemokine that is produced in response to IFN-γ. CXCL10 is important for the recruitment and activation of lymphocytes, neutrophils and NK cells [17]. As a pro-inflammatory chemokine, CXCL10 was found to be closely associated with multiple inflammatory diseases such as immune dysfunction, infectious diseases and tumor development [18]. It is worth to mention, CXCL10 was reported to be correlated with the severity and progression of COVID-19 recently [19–21]. Prior studies have also shown that CXCL10 has predictive ability for sepsis in human adults, infants and neonates [22–24]. To the best of our knowledge, however, there has been little discussion about the association between CXCL10 in serum and the severity of MPP in children. Consequently, we carried out this study and found that the levels of IL-10 and CXCL10 in severe MPP group were both significantly elevated. However, CXCL10 has better specificity in identifying severe MPP, according to the results of ROC analysis. In addition, the level of CXCL10 in serum was associated with the severe MPP-related clinical characteristics, such as duration of fever, length of stay, CRP, LDH, ALT, AST levels and neutrophils proportion in white blood cells. Therefore, we presume that CXCL10 may be a potentially important biomarker to evaluate the progression of MPP and its diagnosis value seems to be greater than that of IL-10. These findings may help pediatricians to make early diagnosis and treatment of severe MPP thereby improving the outcome of children at high risk.

The underlying mechanism of the increased serum level of CXCL10 in children with severe MPP may be related to the pathogenesis of this disease. MPP is typically mild and characterized by fever and a persistent dry cough [25]. However, sorts of cross-reactive antibodies may generate during MP infection due to its extensive homologous sequences compared with mammalian tissues. It may induce autoimmune disorders that involve multiple organ systems, including lung, liver, brain, kidney and smooth muscle [26]. CXCL10 is an IFN-γ inducible chemokine and reacts with its receptor, chemokine (C-X-C motif) receptor (CXCR) 3, which is mainly expressed on Th1 cells [27]. Hence, CXCL10 is most often correlated with Th1-mediated inflammatory disorders. Recently, it has been shown that CXCL10 plays a vital role in autoimmune disease development, such as inflammatory bowel disease and type 1 diabetes through enhancing the Th1 autoimmune response [28]. CXCL10 is also strongly upregulated in many other autoimmune and inflammatory diseases like multiple sclerosis and allograft rejection, resulting in the accumulation of CXCR3^+^ T cells [29, 30]. Hence, the elevated level of CXCL10 may indicate the development of autoimmune inflammation because of antigen cross-reactions, which may account for the exacerbation of MPP.

Our study has several limitations worth discussing. First, this is an observational single-center study with a relatively small sample size. Second, considering that CXCL10 is induced by IFN-γ, it is not yet clear if the increased level of CXCL10 is associated with IFN-γ or other cytokines levels in severe MPP children. Further work is required to validate our findings and better understand the mechanisms underlying the increased level of CXCL10 in severe MPP.

In conclusion, CXCL10 may act as a potential indicator for severe MPP in children. The precise role of CXCL10 in the pathophysiological process of MPP waits for further investigations to elucidate its clinical implications.

## Data Availability

The datasets used and analyzed during the current study are available from the corresponding author on reasonable request.

## Abbreviations

MP: *Mycoplasma pneumonia*
MPP: *Mycoplasma pneumoniae* pneumonia
AUC: Area under the curve
CAP: Community-acquired pneumonia
ROC: Receiver operating characteristic
CRP: C-reactive protein
LDH: Lactate dehydrogenase
ALT: Alanine aminotransferase
AST: Aspartate aminotransferase
WBC: White blood cell count
MALP-2: Macrophage-activating lipopeptide 2
IP-10: IFN-γ-inducible protein-10
Th1: Type 1 T helper
CXCR: Chemokine (C-X-C motif) receptor
